# Duodenal tropism of SARS-CoV-2 and clinical findings in critically ill COVID-19 patients

**DOI:** 10.1007/s15010-022-01769-z

**Published:** 2022-02-18

**Authors:** Michael Neuberger, Achim Jungbluth, Michael Irlbeck, Florian Streitparth, Maria Burian, Thomas Kirchner, Jens Werner, Martina Rudelius, Thomas Knösel

**Affiliations:** 1 Department of General, Visceral and Transplantation Surgery, University Hospital Ludwig-Maximilian-University (LMU) Munich, Marchioninistraße 15, 81377 Munich, Germany; 2 Department of Pathology, Memorial Sloan Kettering Cancer Center (MSKCC), New York, NY USA; 3 Department of Anesthesiology, University Hospital LMU Munich, Munich, Germany; 4 Department of Radiology, University Hospital LMU Munich, Munich, Germany; 5 Institute of Pathology, Ludwig-Maximilians-University (LMU), Munich, Germany

**Keywords:** COVID-19, Duodenitis, SARS-CoV-2, Critically ill patients, Immunohistochemistry, Viral replication

## Abstract

**Purpose:**

Duodenal involvement in COVID-19 is poorly studied. Aim was to describe clinical and histopathological characteristics of critically ill COVID-19 patients suffering from severe duodenitis that causes a significant bleeding and/or gastrointestinal dysmotility.

**Methods:**

In 51 critically ill patients suffering from SARS-CoV-2 pneumonia, severe upper intestinal bleeding and/or gastric feeding intolerance were indications for upper gastrointestinal endoscopy. Duodenitis was diagnosed according to macroscopic signs and mucosal biopsies. Immunohistochemistry was performed to detect viral specific protein and ACE2. In situ hybridization was applied to confirm viral replication.

**Results:**

Nine of 51 critically ill patients (18%) suffering from SARS-CoV-2 pneumonia had developed upper GI bleeding complications and/or high gastric reflux. Five of them presented with minor and four (44%) with severe duodenitis. In two patients, erosions had caused severe gastrointestinal bleeding requiring PRBC transfusions. Immunohistochemical staining for SARS-CoV-2 spike protein was positive inside duodenal enterocytes in three of four patients suffering from severe duodenitis. Viral replication could be confirmed by in situ hybridization.

**Conclusion:**

Our data suggest that about 8% of critically ill COVID-19 patients may develop a severe duodenitis presumably associated with a direct infection of the duodenal enterocytes by SARS-CoV-2. Clinical consequences from severe bleeding and/or upper gastrointestinal dysmotility seem to be underestimated.

**Supplementary Information:**

The online version contains supplementary material available at 10.1007/s15010-022-01769-z.

## Introduction

Severe acute respiratory syndrome coronavirus 2 (SARS-CoV-2) may cause severe coronavirus disease 2019 (COVID-19) involving not only the lungs but, to a varying extent, also other organ systems [1]. The most commonly reported symptoms at the onset of COVID-19 refer to the respiratory tract including fever, cough, and dyspnea [2, 3]. Compared to other critically ill patients, patients with severe COVID-19 also are at an increased risk of developing gastrointestinal symptoms such as diarrhea, nausea, or vomiting [3, 4]. Moreover, gastrointestinal complications were more frequently in critically ill COVID-19 patients than in matched ARDS patients without COVID-19 [5]. Recent data suggest that outcome may be worse if SARS-CoV-2 has also affected the gastrointestinal tract [6–9].

The specific type of gastrointestinal complication varies depending on site of infection by SARS-CoV-2. Up to 50% of the patients may develop hypomotility-related disorders of variable severity, and about half of these patients may suffer from gastric feeding intolerance due to high gastric residuals requiring the insertion of a jejunal feeding tube. Furthermore, a portion of these COVID-19 patients may present with gastrointestinal bleeding due to erosions and ulcers [10]. In addition, there are increasing reports of perforated duodenal ulcers in critically ill COVID-19 patients, although a direct association with SARS-CoV-2 has not yet been confirmed [11–13].

Affection of the gastrointestinal tract by SARS-CoV-2 involves the major receptor of SARS-CoV-2 spike proteins and angiotensin-converting enzyme 2 (ACE2), which is expressed in cells of the gastrointestinal tract in large quantity [7, 14, 15]. Spike protein thus plays an important role in virus pathogenesis and transmission, making it an important target for currently available therapeutics and vaccines and can be used as a diagnostic marker in tissue [16].

Moreover, it is known that approximately 50% of patients with COVID-19 have detectable viral RNA in the stool, and SARS-CoV-2 nucleocapsid protein/RNA has been detected in epithelial cells of the stomach and the rectum [4, 17].

So far, SARS-CoV-2 involvement of the duodenum and its relevance for critically ill patients with COVID-19 has not yet been studied in detail, since most research so far has not focused on this organ. In the present case series, we report about critically ill patients with severe COVID-19 disease who had required an endoscopy for placing a jejunal feeding tube or for endoscopic bleeding diagnosis. Routine mucosal biopsies taken to further clarify the origin/nature of an accompanying duodenitis gave us the opportunity to specifically examine a duodenal enterocyte infection by SARS-CoV-2.

## Methods

### Study design and patients

In this retrospective single-center study, we included critically ill, mechanically ventilated patients, who had been treated from March 04 to June 06, 2020 on a specialized intensive care unit of our institution. All patients had had a severe COVID-19 pneumonia, and had required an endoscopy because of various upper GI complications. SARS-CoV-2 infection had been laboratory-confirmed in all cases by pharyngeal swab specimens using real-time reverse transcription PCR (RT-PCR) for SARS-CoV-2. Subsequently, SARS-CoV-2 RNA had been identified in endotracheal secretion and blood. The diagnosis of COVID-19 had been based on the WHO interim guidance and new coronavirus pneumonia prevention and control programs [18]. Routinely, all patients were screened for other infectious germs. For this purpose, blood serum, endotracheal secretions, and bronchoalveolar lavage (if available) were microbiologically tested for common and relevant viruses, bacteria, and fungi on a regular basis.

Adjuvant systemic therapy had followed internationally recommended standards. All patients had received a stress ulcer prophylaxis by proton pump inhibitors, and anticoagulants according to international recommendations.

The anonymous retrospective data analysis was approved by the local institutional review board (#20-763).

### Computed tomography

Abdominal multislice computed tomography (MS-CT) in venous contrast phase was performed for diagnostic reasons in patients presenting with unclear abdominal symptoms.

### Endoscopy and diagnosis of duodenitis

Indication for a gastroduodenal endoscopy was (a) a severe gastric feeding intolerance which had been resistant to prokinetic treatment, and had necessitated the insertion of a jejunal tube for post-pyloric enteral nutrition, and (b) major gastrointestinal bleeding which had requiring the transfusion of PRBCs, and an endoscopic diagnosis to evaluate the source of bleeding.

Duodenitis was diagnosed in those patients who presented with edema or bleeding erosions of varying extent at the duodenum. In case of a duodenitis, we recorded endoscopic images of the site of the lesion, and we took biopsies from suspected duodenal mucosa to identify the underlying mechanisms of the morphologic changes. Biopsies were immediately placed into formalin for histopathological examination.

### Immunohistochemistry and in situ hybridization

We used commercially available mAbs to SARS-CoV-2 antigens characterized by their claimed specificity for defined viral antigens (nucleocapsid NP, spike protein, S1/S2 subunit). As previously described [19], we focused on two established monoclonal reagents (Sinobiologicals to NP, clone 001, and Genetex to S2 subunit spike protein, clone 1A9). Immunohistochemical staining was performed on a Leica Bond-3 (Leica) platform as described before [20]. A polymeric secondary kit (Refine, Leica) was used for the detection of the primary. Controls were included in our study with a control multi-tissue block (Supplementary Fig. 1). Immunoreactivity was tested employing multi-tissue blocks of formalin-fixed paraffin-embedded pellets of commercially available HEK293 cells transfected with various SARS-CoV-2 proteins (RayBiotech, Peachtree Corner, GA) as previously described [19]. As positive control for mAb 1A9 HEK293, cells transfected with the S2 subunit of the SARS-CoV-2 spike protein were used. Pellets of transfected HEK293 cells expressing nucleoprotein and S1 spike protein subunit served as negative controls. All antibodies were tested at various concentrations ranging from 0.1 μg/ml to 10 μg/ml. Different antigen retrieval steps were employed comprising the heating of samples in low pH (ER1, Leica) or high pH (ER2, Leica) retrieval buffer solution or enzymatic digestions (Enzyme 1, 10’; Leica). For ACE2 immunohistochemistry, we used commercially available mAb (Abcam, ab108252), and we deparaffinized 2 µm formalin-fixed and paraffin-embedded tissue sections. Antigen retrieval was performed by cooking in pH6 for 20 min in the microwave. After incubation with the primary antibody for 1 h, detection was completed with an Impress kit (Vectastain) according to the manufacturer’s protocol.

For in situ hybridization, we used 2 µm formalin-fixed and paraffin-embedded tissue sections. Hybridization with sSARS-CoV-2 sense and antisense probe (ACD) was carried out according to the manufacturer’s protocol. For detection, OPAL 570 was used. Slides were scanned with Vectra Polaris scanner and multispectral images were acquired.

Coinfection of the duodenal enterocytes with herpesviruses like cytomegalovirus (CMV) or herpesvirus (HSV) was excluded by performing standard immunohistochemical staining of the duodenal biopsies with CMV and HSV-1 and HSV-2 antibodies (Supplementary Fig. 2). The clones of the antibodies used were PDM075R9 (Diagnostic BioSystems) for CMV and PDRM001 (Diagnostic BioSystems) for HSV 1 and 2.

### Statistical analysis and data collection

Data on clinical/histopathological findings were extracted from specific local databases used for patient management. Continuous variables were expressed as median and IQR (inter-quartile range). Categorical data were presented as numbers (%).

## Results

### COVID-19 collective at ICU (intensive care unit)

During the observation period, we treated 51 critically ill patients suffering from severe COVID-19 pneumonia. Median ICU LOS (length of stay) was 19 (0–74) days, and hospital mortality was 25.5%.

### Feeding intolerance and gastroscopic findings

Among those 51 patients, nine (18%) male patients developed a feeding intolerance in median 21 days after ICU admission (IQR: 26–14 days). All patients were free of gastrointestinal symptoms at the beginning of COVID-19, but developed a gastric reflux of more than 1000 ml per day during ICU stay. Two of those nine patients also sustained a severe upper gastrointestinal bleeding requiring the transfusion of a large number of PRBCs (packed red blood cell) (42 and 20 units during ICU stay). Bleeding source was a diffuse mucosal bleeding in the inflamed duodenum not amenable to an endoscopic therapy. A localized bleeding ulcer could not be found. Bleeding stopped by conservative therapy after pausing anticoagulation, and after implementation of an aggressive procoagulatoric and proton pump inhibitor therapy. Anti-acidic therapy included a continuous infusion of pantoprazole (8 mg/h), procoagulatory therapy aimed at the maintenance of an INR < 1.2 and a thrombocyte count > 100 G/L.

**Fig. 1:**
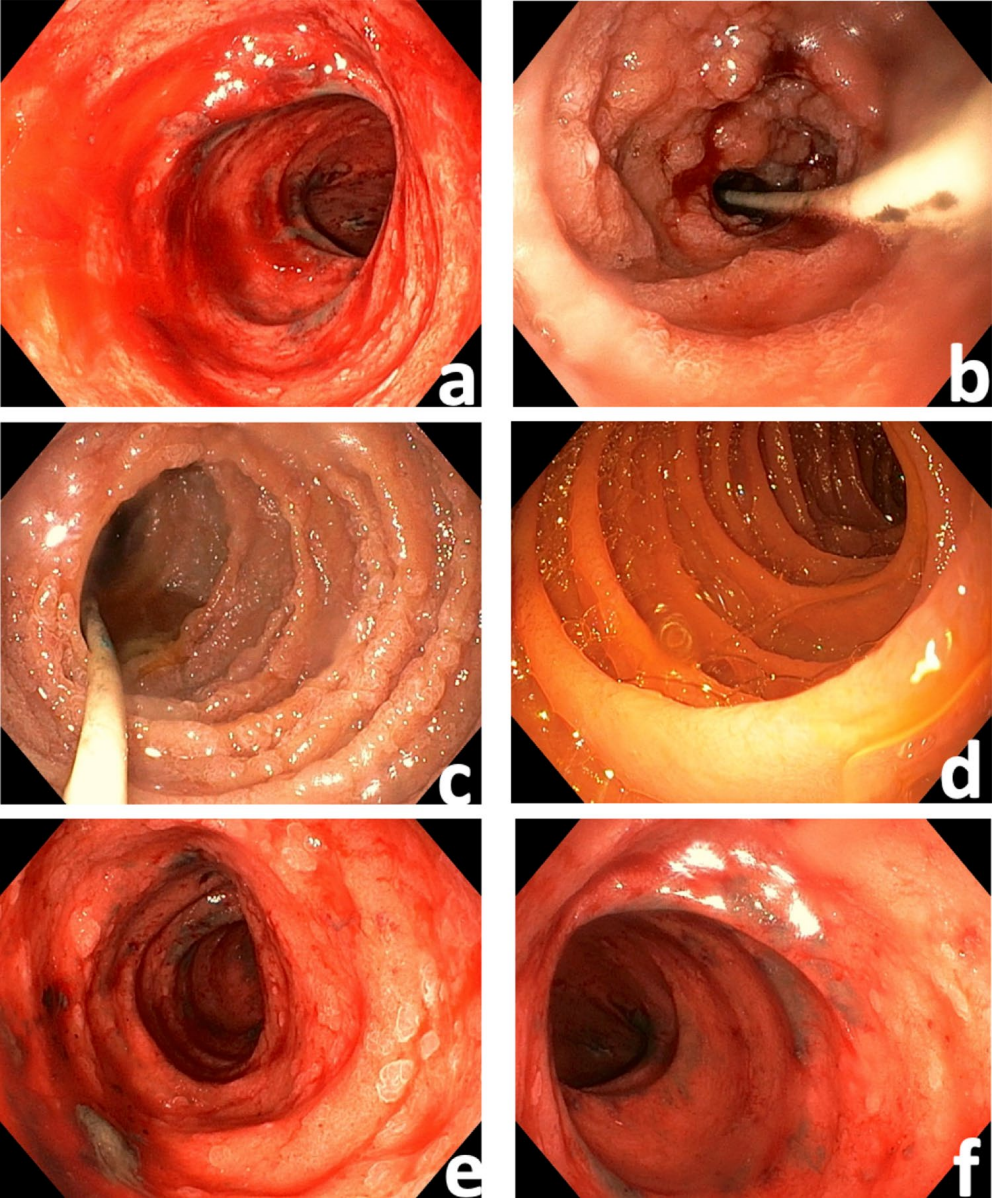
Abnormal endoscopy findings at the duodenum in critically ill patients with SARS-CoV-2 pneumonia presenting with severe upper GI motility disorders. **A**: Diffuse bleeding and mucosal edema. **B** and **C**: Severe inflammation with diffuse bleeding erosions at the duodenal flexure area. **D**: In the distal part of the duodenum, mucosa seems to be macroscopically normal. Another patient with inflamed mucosa and “cobblestone relief” in the upper part of the duodenum (**E**), whereas in the distal part (**F**), inflammation is less marked

Five patients had signs of a minor duodenitis, whereas the remaining four patients (44%) presented with a severe edematous and erosive duodenitis (Fig. 1) 25 (23–34) days after the diagnosis of SARS-CoV-2 infection had been made.

**Fig. 2:**
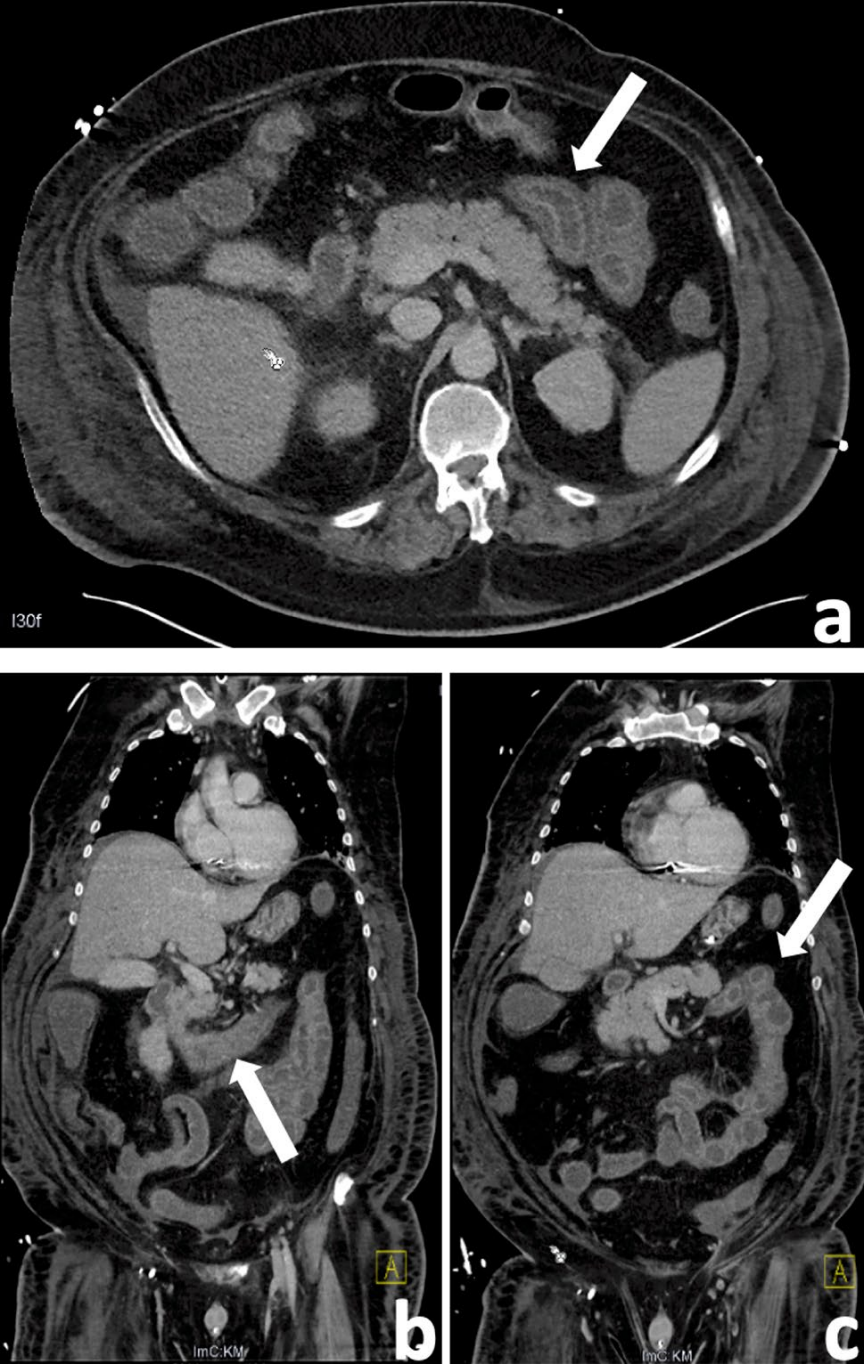
Abdominal contrast-enhanced CT findings of a 48-y male patient with COVID-19 presenting with duodenitis. Axial (**A**) and coronal (**B**, **C**) views show a marked edematous wall thickening in both the proximal and less marked in the ascending duodenum (arrow) and jejunum, while the descending duodenum shows no signs of inflammation, corresponding to the endoscopic finding

No patient had signs of erosive gastritis. Duodenitis was mainly evident in the proximal part of the duodenum, whereas in the more distal parts, inflammation was less marked. Abdominal contrast-enhanced CT scans confirmed this finding in all four cases (Fig. 2).

All patients with a severe duodenitis were male; BMI was 36 (28–43) kg/m^2^ and age at the time of ICU admission was 56 (46–73) years. Maximum APACHE- and SOFA-Score during ICU stay was 45 (39–49) and 18 (15–21), respectively.

Clinical characteristics of these patients are shown in Table 1. Six (67%) of the nine patients presenting with a feeding intolerance, and two of the four patients presenting with a severe duodenitis died on the ICU because of progressive multi-organ dysfunction (uncontrollable respiratory failure). At the time of initiation of invasive ventilation, the corona viral load (RNA copy number) was 10.170.000 K/mL (297.500–55.000.000) in endotracheal secretions, and < 5.600 K/mL in serum in all patients. At diagnosis of duodenitis by endoscopy, viral load decreased to < 5.600 K/mL in endotracheal secretions in all but one patient who still had 670.000 K/mL. In serum, virus RNA was no longer detected at this timepoint in any of the patients.

**Table 1:** Demographics, baseline variables and clinical outcomes of critically ill SARS-CoV-2-positive patients with severe duodenitis

Patient	#1	#2	#3	#4
Demographics				
Age (years)	65	46	75	47
Gender	male	male	male	male
BMI (kg/m^2^)	28	43	28	43
Blood group, Rhesus factor	A +	0-	A +	0 +
Coexisting illness				
Obesity		Yes		Yes
Hypertension	Yes		Yes	Yes
Diabetes mellitus			Yes	Yes
Cardio-cerebrovascular disease			Yes	Yes
Chronic lung disease		Yes		
Chronic kidney disease				Yes
Duodenitis verification data				
Gastroscopy indication	Reflux	Reflux, bleeding	Reflux	Reflux, bleeding
Severe Duodenitis (endoscopic, histological)	Yes	Yes	Yes	Yes
Identification of virus spike protein (IHC)	No	Yes	Yes	Yes
Time from ICU admission to duodenitis (days)	25	35	22	19
Temperature at endoscopy (°C)	37.7	39.6	38.1	37.5
Horowitz-Score at endoscopy	300	196	214	110
SOFA-Score at endoscopy	16	14	13	18
APACHE II-Score at endoscopy	33	34	37	39
Clinical course during ICU stay				
Time from COVID-19 diagnosis to intubation (days)	2	1	4	5
Duration of mechanical ventilation (days)	67	46	45	27
ECMO-Therapy	No	No	No	No
Days from positive to negative virus test results	26	31	43	-
Length of Hospital stay (days)	76	57	54	29
Organ failure during ICU stay				
Septic shock	Yes		Yes	Yes
Acute kidney failure requiring renal replacement therapy	Yes	Yes	Yes	Yes
Acute liver failure	Yes			Yes
Myocardial infarction/CPR	Yes		Yes	
Secondary lung fibrosis			Yes	
Pulmonary superinfection^a^		Yes		
Additional gastrointestinal symptoms^b^	Yes			Yes
SOFA-Score (maximum during ICU stay)	22	16	15	19
APACHE II-Score (maximum during ICU stay)	43	38	46	50
Clinical outcome	Died	Discharged	Discharged	Died

*IHC* immune-histochemical verification

^a^Candida, Mycoplasma, HSV-1, CMV

^b^Other than duodenitis: paralytic ileus, pancreatitis, and pancolitis

### *Histopathological, immunohistochemical, and *in situ* hybridization findings in the duodenum*

**Fig. 3:**
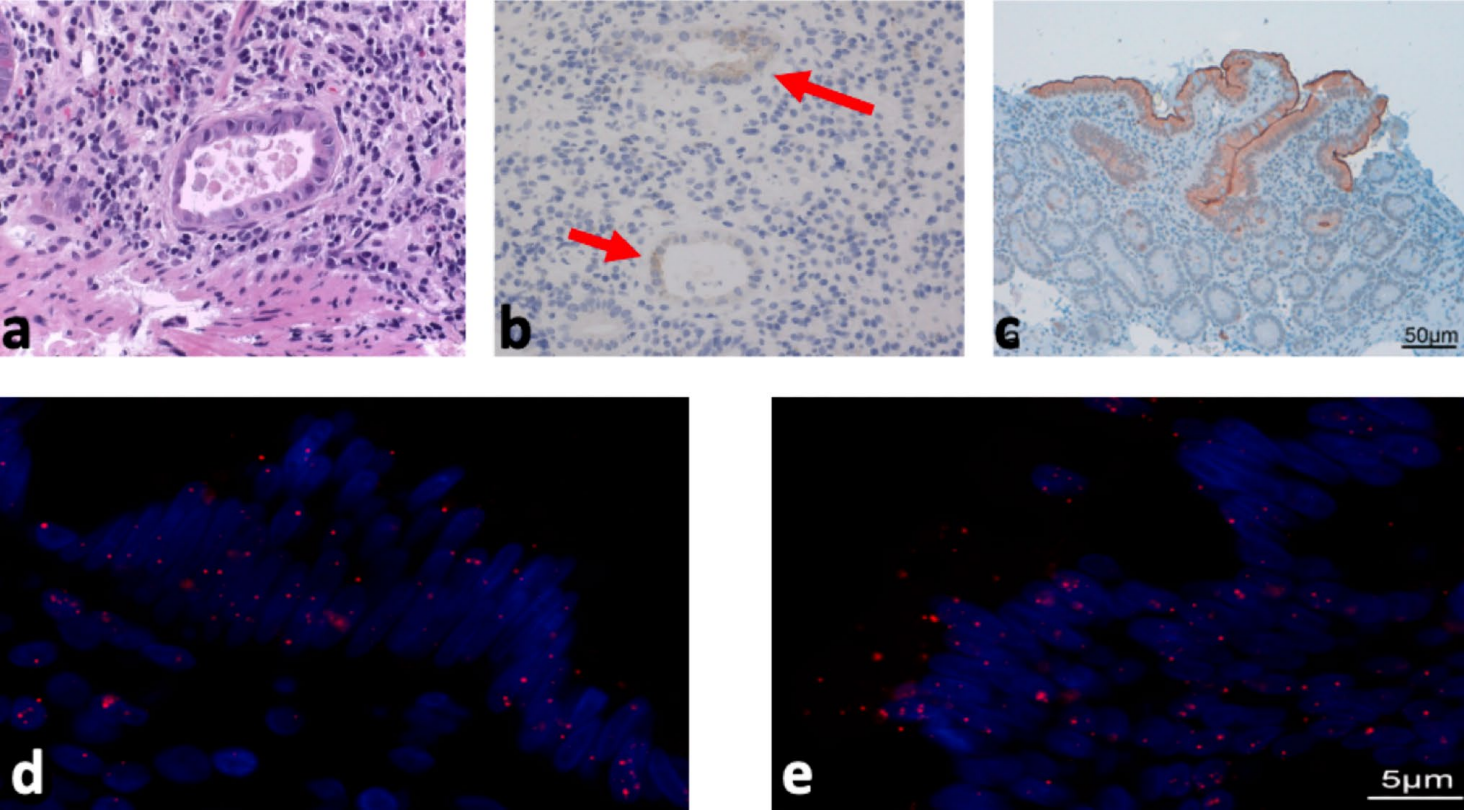
**A** Duodenal crypt with small acinus, and with intracytosolic and intranuclear inclusions with halo and apoptosis consistent with a viral infection (H&E 20 × magnification). **B** Positive immunohistochemical expression of the SARS-CoV-2 spike protein in these duodenal crypts (arrows, 20× magnification, COVID-19-S2-Subunit of the spike protein, clone 1A9). **C** Immunohistochemistry showing that ACE2 is expressed in enterocytes with strong expression on the cell surface. In situ hybridization of antisense s-SARS-CoV-2 (**D**) and sense s-SARS-CoV-2 (**E**) confirms viral tropism and replication in duodenal biopsies. Viral RNA is visualized as red dots in the cytoplasm and nucleus of enterocytes. Blue staining represents nuclear DNA counterstaining of enterocytes with DAPI

In hematoxylin–eosin stained sections of all four patients with a severe duodenitis, we identified duodenal crypts with small acini and intracytosolic and intranuclear inclusions consistent with a viral infection (Fig. 3A). Immunohistochemically, we detected expression of the SARS-CoV-2 spike protein in these duodenal crypts showing a distinct positive expression in three of the four duodenitis patients (Fig. 3B). In the remaining patient, the biopsy had been taken at a time when pharyngeal swab specimens, endotracheal secretions, and blood had already been negative for SARS-CoV-2.

No patient revealed a positive expression when using the NP antibody. Immunohistochemistry showed a duodenal intranuclear ACE2 receptor expression in all four patients with a severe duodenitis, but also—to the same extent—in a virus negative control sample (Fig. 3C). Specific expression of CMV, HSV 1 or 2 in the biopsies could be excluded by immunohistochemistry (Supplementary Fig. 2).

In situ hybridization of antisense s-SARS-CoV-2 and sense s-SARS-CoV-2 confirmed viral tropism and replication in duodenal biopsies. Viral RNA was visualized as red dots in the cytoplasm and nucleus of enterocytes. DAPI counterstaining (blue color) was used to visualize nuclear cell DNA of enterocytes (Fig. 3 D/E).

## Discussion

Although COVID-19 primarily affects the respiratory tract, emerging data implicate an involvement of the gastrointestinal tract [21–23]. Underlying reason may be primarily an indirect mechanism acting via the enhanced, SARS-CoV-2-related intestinal translocation of toxins and bacteria. SARS-CoV-2-related intestinal microcirculatory failure may alter the gut microbiome, and enlarge the intercellular space between enterocytes. Enhanced translocation may propagate a systemic cytokine storm due to the activation of a great amount of immune-competent cells, common for corona virus infection [24, 25].

Our data suggest that, in addition of those indirect effects, SARS-CoV-2 may also cause direct damage at the intestinal mucosa. Among our 51 critically ill COVID-19 patients, 9 (18%) required endoscopic placement of a jejunal feeding tube due to high gastric reflux and feeding intolerance. During the insertion, we detected a severe erosive duodenitis in four patients (44%). In three of those four patients, we could demonstrate a duodenal SARS-CoV-2 infection. Furthermore, in situ hybridization of antisense s-SARS-CoV-2 and sense s-SARS-CoV-2 confirmed viral tropism and replication in the enterocytes of duodenal biopsies. To date, although there are data that could detect virus in small-bowel tissue, replication of SARS-CoV-2 has been demonstrated mainly in animal models [26]. Interestingly, SARS-CoV-2 RNA could not be detected in biopsies of the five patients with a mild duodenitis but only in patients with the severe progressive form. Therefore, an SARS-CoV-2-associated severe duodenitis/duodenal SARS-CoV-2 infection may occur in about 6–8% of critically ill COVID-19 patients.

Two of our four patients with a severe duodenitis also had diffuse, severe upper gastrointestinal bleeding from duodenal erosions not amenable to an endoscopic therapy. Gastrointestinal hemorrhage has been found in the literature in 2–13% of hospitalized COVID-19 patients, with bleeding sources mostly localized in the esophagus, stomach, and lower gastrointestinal tract [4, 17, 27]; in many cases, however, the exact cause of bleeding could not be determined because of a conservative treatment forgoing a diagnostic endoscopy [28].

A recent study characterized bleeding etiologies in 41 COVID-19 patients. Unlike our two ICU patients who presented with diffuse erosive duodenal bleeding, this study identified localized peptic gastric or duodenal ulcers as the most common etiology (80%) for upper gastrointestinal bleeding [29]. However, our observations are in line with the finding that bleeding occurs comparably late during ICU stay, rather than early after ICU admission, suggesting a secondary infection of the duodenum as bleeding origin.

A recently published international multicenter study of COVID-19 patients that focused on endoscopy detected gastroduodenal ulcers in almost 30% of upper gastrointestinal endoscopies, of which nearly 64% were actively or recently bleeding, and diffuse erosive damage in 15% [30]. The mucosal changes found were quite similar to those in our collective, which, however, included only critically ill patients requiring intensive care. Nevertheless, Vanella et al. stated in their investigations that discovered severe gastrointestinal mucosal damage was not dependent on preexisting conditions [30]. It should be noted that our critically ill patients had comorbidities known to be risk factors for severe and fatal disease progression, including higher age, obesity, diabetes, and hypertension, as well as chronic organ dysfunctions predominantly affecting the heart, liver, and kidneys [31–33]. However, whether these risk factors had an influence on the gastrointestinal course of our patients cannot be concluded on the basis of the small collective.

Although gastrointestinal manifestation of COVID-19 could also be attributed to pharmacological adverse events or metabolic disturbances in critically ill patients, a direct infection of enterocytes is possible [10]. Expression of angiotensin-converting enzyme 2 (ACE2), the major receptor of SARS-CoV-2, not only occurs in the heart, kidneys and alveolar cells of the lungs, but also in glandular epithelial enterocytes of the upper esophagus, ileum, and colon/rectum [14, 17, 34–37].

A recent study evaluating all major organs post-mortem by autopsy found ultrastructural aggregates of viral particles in gastric enterocytes. Post-mortem autolysis, however, prevented a thorough investigation of the gastrointestinal system and assessment by SARS-CoV-2 immunohistochemical staining [1]. An in-vitro model using human small intestinal enteroids, including the duodenum, demonstrated a productive infection of SARS-CoV-2 [38]. In a hamster model, immunohistochemistry assays demonstrated viral antigens in epithelial cells of the duodenum on days 2 and 5 after inoculation with SARS-CoV-2 [39].

Although there are increasing reports of severe upper gastrointestinal bleeding and complications, thus far, there are only few in vivo reports that mention the human duodenum as a potential concomitant manifestation of SARS-CoV-2 infection. Lin et al. examined duodenal swabs of six unselected COVID patients presenting with severe gastrointestinal symptoms, and identified SARS-CoV-2 RNA in two patients. Safari et al. obtained tissue samples during emergent laparotomy for a perforated peptic ulcer and found SARS-CoV-2 RNA in the duodenal wall of a COVID-19 patient [4, 40]. The clinical significance of these findings, however, is unclear.

Our in vivo results suggest that in critically ill COVID patients, there may be a strong association between a duodenal SARS-Cov-2 infection, a severe duodenitis, and subsequent bleeding complications. Observations that SARS-CoV-2 infection can be associated with severe endothelialitis and multiple microthrombi in the venous vascular bed of the large bowel support our hypothesis in the duodenum [41]. Moreover, a recent case report identified microthrombosis in the duodenum of a COVID-19 patient with gastrointestinal bleeding [42].

Findings of plasma cells and lymphocytes in lamina propria of the duodenum underline microvascular small-bowel injuries, likely contributing to severe disease progression [26].

The fact that we were unable to detect SARS-CoV-2 RNA in one patient with a severe duodenitis may relate to the timing of the endoscopy. At the time of the biopsy, serum samples and pharyngeal swabs of this patient were already SARS-CoV-2 negative, so it might have been too late to detect virus protein although duodenitis was still present. According to this, the previous findings demonstrated that gastrointestinal infection sometimes last even after viral clearance in the respiratory tract, implicating a time shifting course of the stage of viral inflammation and clearance [17].

Altogether, our observations support the hypothesis that a severe duodenitis is a consequence of direct enterocyte infection by SARS-CoV-2 rather than an unspecific immunological consequence of generalized COVID-19 disease. Most likely, CoV-2 infection of the gastrointestinal tract is secondary resulting from swallowing of virus-containing pulmonary expectorate (before intubation), or from hematogenic spread (second hit hypothesis) as proposed before [24].

Direct SARS-CoV-2 affection of the duodenum (6–8% in our report) appears to be an additional manifestation of COVID-19 standing in a line with affection of the pulmonary system, of the cardiovascular system (incidence 20–60%), of the hepatocellular system (14–53%), of the renal system (up to 31%), and of the gastrointestinal tract as a whole (12–61%) [43].

## Conclusion

Our data suggest that SARS-CoV-2 can also infect duodenal enterocytes leading to severe duodenitis; the duodenum might be an underestimated infection site potentially associated with severe bleeding complications. When assuming that COVID-19 patients without a severe upper gastrointestinal motility disorder do also not suffer from a severe SARS-CoV-2 duodenitis, it can be estimated that the latter symptom occurs in 6–8% of critically ill COVID-19 patients.

To our knowledge, this study focus for the first time on the duodenum in critically ill COVID-19 patients, thus mentioning the involvement of this organ as an underestimated complication in this patient group.

## Supplementary Information

Below is the link to the electronic supplementary material.

Supplementary Fig. 1: A Composite positive–negative control as part of the multi-tissue block in 5 × magnification. In the center brownish stained cell line tissue infected with 1A9 SARS-CoV-19 can be seen as a positive control. Surrounding it, stained bluish, is lymphoid tissue as a negative control. B Picture shows transfected HEK293 cells transfected with the S2 subunit of the SARS-CoV-2 spike protein in 20 × magnification, serving as positive control for mAb 1A9

Supplementary Fig. 2: Standard immunohistochemical staining of duodenal biopsies with CMV (A) and HSV 1 and HSV 2 (B) antibodies showing no specific expression of coinfection with these herpes viruses
