# Supplementary figures and images for: Duodenal tropism of SARS-CoV-2 and clinical findings in critically ill COVID-19 patients
Source: Infection. 2022 Feb 18;50(5):1111–20. doi: 10.1007/s15010-022-01769-z (PMC8857399; doi:10.1007/s15010-022-01769-z)

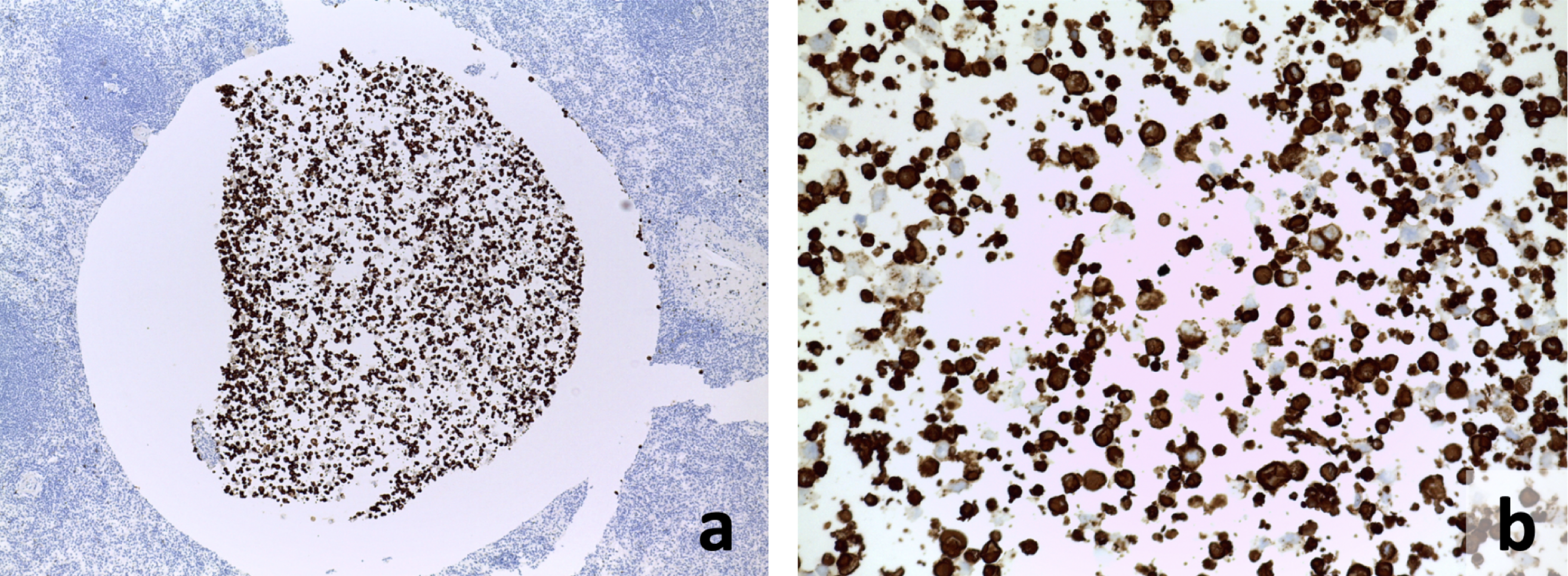

Supplement: Supplementary file 1 — Supplementary Fig. 1: A Composite positive–negative control as part of the multi-tissue block in 5 × magnification. In the center brownish stained cell line tissue infected with 1A9 SARS-CoV-19 can be seen as a positive control. Surrounding it, stained bluish, is lymphoid tissue as a negative control. B Picture shows transfected HEK293 cells transfected with the S2 subunit of the SARS-CoV-2 spike protein in 20 × magnification, serving as positive control for mAb 1A9 [file 15010_2022_1769_MOESM1_ESM.tiff]

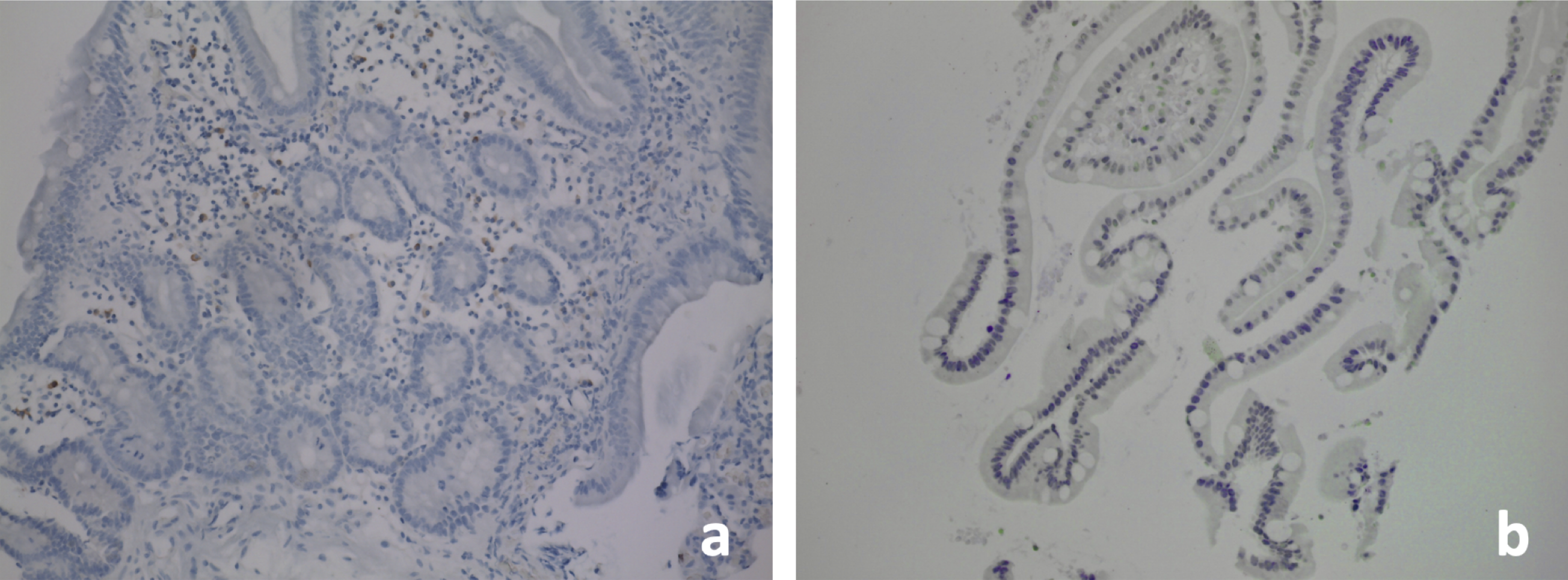

Supplement: Supplementary file 2 — Supplementary Fig. 2: Standard immunohistochemical staining of duodenal biopsies with CMV (A) and HSV 1 and HSV 2 (B) antibodies showing no specific expression of coinfection with these herpes viruses [file 15010_2022_1769_MOESM2_ESM.tiff]
